# Quality of care of patients with acute myocardial infarction in Bulgaria: a cross-sectional study

**DOI:** 10.1186/1472-6963-9-15

**Published:** 2009-01-26

**Authors:** Milka Ganova-Iolovska, Krassimir Kalinov, Max Geraedts

**Affiliations:** 1 National Center of Public Health Protection, 15, Ivan Ev. Geshov Blvd, 1341 Sofia, Bulgaria; 2 New Bulgarian University, Department of Computer Science, 21, Montevideo Street, 1618, Sofia, Bulgaria; 3 Institute for Health Systems Research, University of Witten/Herdecke, Alfred-Herrhausen-Str. 50, 58448 Witten, Germany

## Abstract

**Background:**

Cardiovascular diseases are the major cause of death in Bulgaria. Because of notable differences in mortality rates between Bulgaria and other European countries, we presume a tangible difference in the management of acute myocardial infarction (AMI) and an underutilization of evidence-based treatments. In order to determine the quality of care of patients with AMI in Bulgaria, we analyzed the appropriateness of current treatments and their relation to patient characteristics.

**Methods:**

We performed a descriptive cross-sectional study, using retrospectively collected data from medical charts. We included all patients with AMI, residing and admitted to hospitals in the region of Stara Zagora, Bulgaria, between September 1st and December 31st, 2004. Socioeconomic status was surveyed within the framework of a structured patient interview. We used chi-square tests with Fisher's exact probabilities to analyze the relationship between prehospital time delay age, sex, and socio-economic status of the patients and Student's independent samples t-tests to check hypotheses about means.

**Results:**

From 134 patients with AMI (mean age 64.6, SD 13.2, 66% male), 7% presented to a hospital within 59 minutes, and 44% within 4 hours of symptoms onset. The use of Heparin was 98%. In the first 24 hours, ASS was administrated in 82% and β-Blockers in 73% of the cases. At discharge Aspirin, β-Blockers, Angiotensin Converting Enzyme Inhibitors or AR-Blockers and Statins were used in 85%, 79%, 66%, and 43% of cases respectively. Intravenous fibrinolysis was applied in 32% of the eligible patients with ST-segment elevation. Percutaneous coronary interventions were applied in four patients within the first month after AMI. Hospital location in relation to a patient's place of residence and manner of transportation to hospital did not influence the time delay between the onset of symptoms to the start of hospital treatment. In the study region, a relation between time delay and both age and education level was observed.

**Conclusion:**

The actual quality of care of patients with AMI in Bulgaria lies far from the evidence-based recommendations. Additional research and improvements in health services are needed to reduce the burden of cardiovascular disease in Bulgaria.

## Background

As in many parts of the world, cardiovascular diseases (CVD) are the main cause of death and disability in Bulgaria. CVD were responsible for 61.5% of all deaths in Bulgaria in 1990, and for 67.5% of all deaths in 2004 [1,2]. In 2004, 16.7% of all deaths were caused by ischaemic heart diseases (IHD) and 6.4% by acute myocardial infarction (AMI).

Considering the CVD mortality rates in Bulgaria, one of the goals of the health care reforms that replaced the old soviet-style system in Bulgaria was to decrease CVD mortality by implementing evidence-based diagnostic and treatment methods.

During the past 20 years, intravenous fibrinolytic therapy and/or percutaneous coronary intervention (PCI) were introduced as main options for managing AMI, improving mortality rates and reducing recurrent myocardial infarctions [3-6]. PCI may be applied for more advanced stages of AMI, and the application of fibrinolytic therapy in ST-segment elevated myocardial infarction (STEMI) and the benefits thereof are directly associated to the time elapsed from the onset of the first symptoms to the beginning of treatment [3,4,6].

However, some recent international studies indicate that gaps exist between the guidelines of the American College of Cardiology/American Heart Association and the actually performed in-hospital management of acute coronary syndrome (ACS) [7,8].

In order to evaluate the quality of care of ACS patients in Bulgaria, we compared the actual management of AMI patients in a Bulgarian region to internationally accepted evidence-based standards. As main factors which could influence the mortality rates of IHD and AMI we investigated the timing as well as the currently performed treatment approaches of AMI inpatients.

## Methods

### Study region

The survey was carried out in the Stara Zagora region, which is typical for Bulgaria in its demographic (age, sex and urban/rural distribution) and health care characteristics. The region includes nearly 5% (362,100 inhabitants) of the total Bulgarian population of about 7.8 million [9,10].

In-hospital care of patients with AMI is provided in six hospitals of the region. All of them have intensive care units and full-time residents in internal medicine. Five hospitals have full-time residents in cardiology as well. PCI facilities are not available in any of the hospitals in the Stara Zagora region.

In general, the pre-hospital emergency services in Bulgaria are provided by emergency medical facilities (EMF). The EMFs are located in every regional centre as well as in the bigger municipality centres. They provide emergency services (EMS) such as check-ups, medication of symptoms, resuscitation and, in accordance to their judgment, transportation to hospitals. Bulgarian general practitioners (GP) supply EMS to patients on their lists on rare occasions. In some cases, either because of the impossibility of receiving medical care outside the hospital or because of the severity of a patient's condition, the patient is transported with a private carrier to hospital without any prehospital investigation.

In the region of Stara Zagora, EMS are provided by three EMF, one in the regional centre and two in the municipality centres.

### Study population

During the period from September 1st to December 31st of 2004, all inpatients residing in the region who were admitted and treated at any of the six hospitals of the region with the discharge diagnosis AMI (STEMI and NSTEMI), (ICD-IX 401.0-.9) were included in the study. The criteria used in Bulgarian hospitals for the discharge diagnosis of AMI are as follows [11]:

1. STEMI: ischaemic symptoms lasting ≥ 20 min, cardiac marker evidence of myocardial necrosis (positive CK-MB and other markers) and new (or presumably new if no prior electrocardiography (ECG) is available) ST-segment elevation.

2. NSTEMI: cardiac marker evidence of myocardial necrosis (positive CK-MB or troponin) without new ST-segment elevation.

Consent for participation in the study was obtained from hospital boards of managers and from each patient or patient's relatives. Since there are no ethics committees in Bulgaria, the study was approved by the Ministry of Health for its accordance to ethical standards.

### Data from inpatient records

One physician extracted the data from inpatient medical records following patient discharge in a structured data collection form. A second physician performed a review of a random sample of 20% of the records from each hospital. Consistency between the two data sets was 98%. Data entry to a computer database was performed by a qualified person. A second data entry was performed, using a control program warning of data differences.

#### Pre-hospital time delay

The data for calculating the pre-hospital delay of time were extracted from the patients' charts. The pain-to-door time was categorized into six time intervals – up to 0:59 h, from 1:00 up to 1:59 h, from 2:00 up to 3:59 h, from 4:00 up to 11:59 h, from 12:00 up to 23:59 h and 24 h and more. Two patient groups were considered separately in the analysis: patients who presented to a hospital within their area of residence formed one group, while patients who presented to a hospital outside of their area of residence formed a second group. If a patient was transferred from one hospital to another within the same region, only the time of the first hospitalization was considered.

#### Medical history

Reviewing the medical records, we also abstracted information about medical history of previous myocardial infarction (MI), angina pectoris (AP), strokes and co-morbidity (hypertension, diabetes mellitus and dyslipidemia). Data concerning family history, smoking habits and BMI were missing in more than 40% of the medical records and were therefore not included in the study.

#### In-hospital data

ECG findings, treatment approaches and specific contraindications, complications, length of stay (LOS) and in-hospital outcomes were collected from inpatient records.

The ***specific contraindications for administered medication***, clinically documented in the patient's medical record, were defined as follows: for the use of Aspirin: intolerance, allergy, active bleeding, a history of gastrointestinal or genitourinary bleeding, ulcers, dyspepsia, a platelet count of <100,000/mm^3^, anaemia, use of other anticoagulants; for β-Blockers: allergy, hypersensitivity, bradycardia, AV-block greater than I° degree, cardiogenic shock, hypotension, chronic obstructive pulmonary disease, asthma or bronchospasm; for Heparin: active or recent bleeding, a platelet count of <100,000/mm^3^, ulcers or serious gastrointestinal or genitourinary bleeding, a history of known heparin-induced thrombocytopenia, severe co-morbidities; for lipid-lowering drugs: allergy, hypersensitivity, hepatic or renal dysfunction, abnormal liver function test results, primary biliary cirrhosis; for ACE Inhibitors: allergy, intolerance, hypersensitivity, impaired renal function, hypotension, hyperkalemia or liver disease.

Absolute contraindications for fibrinolysis therapy in patients with acute STEMI were defined as follows: any prior cerebral haemorrhage; known structural central nervous system lesion (AV malformation, tumor, etc.); ischemic stroke within 3 months except acute ischemic stroke within 3 hours; significant head trauma within 3 months; suspected aortic dissection; active bleeding or bleeding diathesis (excluding menses). Relative contraindications were: history of chronic, severe, poorly controlled hypertension; severe uncontrolled hypertension on presentation (SBP greater than 180 mm Hg or DBP greater than 110 mmHg); traumatic or prolonged cardiopulmonary resuscitation (greater than 10 minutes) or major surgery (less than 3 weeks); major surgery or internal bleeding within 3–4 weeks; active peptic ulcer disease; pregnancy; current use of anticoagulants; prior exposure (more than 5 days ago) or prior allergic reaction to the fibrinolytic agents.

The ***complications ***during hospital admission were defined as: the occurrence of a new AV-Block II° or III° degree or bundle branch block (BBB), left ventricular insufficiency, ventricular tachycardia or cardiopulmonary resuscitation with or without ventilation.

### Socioeconomic status (SES)

The SES of the study population was surveyed within the framework of a structured patient interview and took place 14 to 28 days after discharge. The SES includes: 1) education level, coded as none, primary, secondary and college or higher education, 2) employment status, coded as employed, unemployed or pensioner; 3) personal monthly income, divided into four groups according to the income levels in Bulgaria; 4) marital status, coded as married or living with partner and single; 5) number of family members, divided into four groups from one family member up to four and more members (Table 1).

**Table 1:** Study population – basic characteristics

**Basic characteristics**	**N (%/SD)**
**Gender**	
male	89 (66%)
female	45 (34%)
**Age groups**	
26 – 64 years	60 (45%)
≥65 years	74 (55%)
**Education**	
primary school or less	58 (49%)
secondary school	46 (39%)
college or high school	14 (12%)
**Employment status**	
unemployed	98 (83%)
employed	20 (17%)
**Personal monthly income**	
≤ 100 BGN*	33 (30%)
100.1 – 200 BGN	57 (52%)
200.1 – 300 BGN	14 (13%)
≥ 300.0 BGN	4 (4%)
unemployed without unemployment benefits	1 (1%)
**Marital status**	
married/partner	74 (63%)
single	44 (37%)
**Family members**	
1 member	28 (24%)
2 members	54 (46%)
3 members	14 (12%)
4 or more members	22 (19%)
**History of:**	
Angina pectoris	78 (58%)
Previous MI	29 (22%)
MI in year 2004	6 (21%)
Stroke	15 (11%)
Hypertension	99 (74%)
Diabetes	32 (24%)
Hypercholesterolemia	11 (8%)
**STEMI**	82 (61%)
Gender	
male	52 (63%)
female	30 (37%)
Age	
median age in years	62.5
mean age in years	62.0 (12.3)
mean age male	58.7 (12.5)
mean age female	66.0 (9.8)

* Bulgarian currency Lev (BGN); 1 BGN = 0.511 €/0.68 US $

The interviewers were trained at the National Centre of Public Opinion. We selected as interviewers local residents from the Stara Zagora region who were not employed in medical institutions. For the specific aim of the study, the interviewers received additional training.

### Statistical analysis

For data description, we used tables of absolute and relative frequencies for nominal (non-metric) data and mean values and standard deviations (SD) for continuous (metric) data. The chi-square test with Fisher's exact probabilities was used to test the hypothesis on the relation between pre-hospital time delay (grouped in five intervals – up to 0:59 minutes, from 1 up to 1:59 h, from 2 up to 3:59 h, from 4 to 11:59 hours and from 12 to 23:59 h) versus age, sex, education, and personal monthly income. Student's independent samples t-test was applied to check hypotheses about means. Statistical package SPSS/PC^® ^was used for all analyses.

## Results

### Study population

Included in the study were a total of 134 patients with AMI (mean age 67 ± 13.2 years), including 82 (61.2%) with STEMI and 52 (38.8%) with NSTEMI, who were consecutively admitted to in-hospital treatment in the study region (Table 1). The age range of the total population lay between 26 and 90 years. The median age was 62 (mean 61.9 ± 13.7) years for men and 71 (mean 69.9 ± 10.2) years for women. From the discharged patients, five refused to participate in the interviews and one patient died in the first days after discharge. Thus, we could not determine their SES. Table 1 depicts the baseline characteristics of the study population.

In all patients, an ECG and CK-MB examinations were performed. Additionally, in 43 cases (32.1% of the total population), troponin I was investigated. The complications during hospital treatment are presented in Table 2. Cardiopulmonary resuscitation with or without ventilation was performed in 16 subjects (12%). Heart insufficiency was noted in the records of 51 subjects. Ejection fractions (EF) of < 55% were computed in 40 of them.

**Table 2:** Hospital complications

**Complications**	**N (%)**
New AV-block II° and III° degree	11 (8%)

New BBB	3 (2%)

Left ventricular insufficiency	51 (38%)

Ventricular tachycardia	11 (8%)

Shock with resuscitation and/or ventilation	16 (12%)

### Pre-hospital time delay

For patients who presented to the hospital showing clinical signs as well as laboratory and ECG indicators of MI and a symptom onset of > 24 hours ago, the medical records concerning symptom onset were often inaccurate and mostly stated the time of symptom onset as "1–2 days ago" or "a few days ago". Since exact time-related data for these patients was insufficient, the median time delay for the whole study population could not be determined. The calculations presented include only inpatients admitted within the first 24 hours from symptoms onset.

Table 3 presents the time interval "pain to door" according to the patients' place of residence and the hospital location. 66% (N = 88) of the patients were admitted to a hospital in their area of residence and 34% (N = 46) to hospitals outside their area of residence. For all inpatients, STEMI and NSTEMI, the median time was 3:15 hours. For STEMI inpatients, the median time was 3:00 hours (mean 4:32 ± 4:25 h).

**Table 3:** Time-delay according to patients' domicile, hospital location and type of MI

**Time-delay "pain to door"**	**Hospital location/Residence**		
	
	**N equal**	**N outside**	**N (%)**
up to 0:59 h	6	3	9 (7%)
STEMI patients	3	3	6
1 to 1:59 h	11	4	15 (11%)
STEMI patients	9	1	10
2 to 3:59 h	22	13	35 (26%)
STEMI patients	13	8	21
4 to 11:59 h	22	14	36 (27%)
STEMI patients	12	8	20
12 to 23:59 h	6	1	7 (5%)
STEMI patients	5	1	6
>24 h	21	11	32 (24%)
STEMI patients	12	7	19

Total	88	46	134 (100%)
STEMI patients	54	28	82 (61%)

The subgroup of 17 STEMI patients treated with fibrinolysis was admitted for inpatient treatment within a median of 2:00 hours (mean 2:12 ± 1:24 h). Only three of them (18%) were admitted within 59 minutes from infarction onset, and eight within the first 120 min. In the first 4 "golden hours", 15 patients (88% of the patients treated with fibrinolysis) were admitted to the hospital. The remaining two patients had a pain-to-door time between 4 and 12 hours.

A total of 103 subjects (77%) arrived at the hospital with an ambulance and one patient was transferred from another health care facility.

The chi-square test with Fisher's exact probabilities was applied for patients admitted to hospital within the first 24 hours of symptom onset. The analysis did not demonstrate a statistically relevant relation of hospital location and patient residence (community centre with hospital, community centre without hospital or village) or of the manner of transportation (with ambulance or private vehicle) to pre-hospital time-delay (p > 0.05). We found a statistically significant relation between pre-hospital time delay and age as well as education level. Patients aged ≤ 64 years and subjects with higher education (college or high school) arrived at hospital significantly later (see Table 4).

**Table 4:** Relation between patients socio-demographic characteristics* and time-delay, and means, standard errors and p-values of differences for the statistical significant relations

**Variables**	df**	**Chi-square value**	**Exact probability**
Gender	4	0.76	0.9601
			
Age	4	9.67	0.0348***

Education level	12	56.29	0.0029***

Personal monthly income	16	16.04	0.4503

**Time delay (in hours)**			

	**Mean**	**Standard error of mean**	**p-value for difference**

Age			

≤ 64	5.83	0.71	
			0.005***
≥ 65	3.47	0.37	

Education level			

low	3.67	0.44	
			0.024***
high	5.45	0.64	

* Patients with pain-to-door time > 24 h and patients transferred from other hospitals are excluded

** df – degree of freedom

*** the relation is statistically significant

### Treatment with Heparin

Heparinization in the acute care period was performed in 119 cases, thus the frequency of Heparin administration was 98.3%. Contraindications were registered in 7 patients' records, and in the records of another 6 subjects data concerning contraindications for Heparin treatment was missing.

### In-hospital medical treatment within the first 24 hours and at discharge

The medication in the first 24 hours and at discharge is depicted in Table 5. In the first 24 hours ASS was prescribed in 82% and β-Blockers in 73% of the eligible cases. At discharge ASS was prescribed in 85% of cases, β-Blockers in 79%, ACE-Inhibitors or AR-Blockers in 66% and lipid-lowering drugs in 43%.

**Table 5:** In-hospital medical treatment in first 24 hours and at discharge*

		95% CI**					
						
**Drugs**	**N (%)**	**Lower (%)**	**Upper (%)**	**Males (total %)**	**Females (total %)**	**% of patients** **≤ 65 yrs**	**% of patients** **≥ 65 yrs**
≤ 24 hours***							

ASS	92 (82%)	70.7	86.8	82%	83%	77%	87%

β-Blockers	72 (73%)	63.1	80.8	72%	75%	71%	74%


**at discharge**							

ASS	95 (85%)	78.5	91.4	84%	86%	79%	90%

β-Blockers	99 (79%)	65.6	82.4	75%	88%	73%	85%

ACE Inhibitors/AR Blockers	78 (66%)	57.6	74.6	62%	75%	64%	68%

Lipid-lowering drugs	52 (43%)	34.4	52.2	35%	54%	47%	40%

* Patients with contraindications and missing values are excluded

** CI – confidential interval

*** Figure excludes patients who died during hospitalisation

Contraindications for ASS were registered in 12 patients, for β-Blockers in 25, for ACE-Inhibitors or AR-Blockers in 6 and for lipid-lowering drugs in 4 patients.

At admission slightly more women were medicated with ASS and β-Blockers than men. ASS was more often prescribed in patients in the age group ≥ 65. At discharge more women received β-Blockers, ACE-Inhibitors or AR-Blockers and lipid-lowering drugs than men. More patients in the age group ≥ 65 years received a discharge medication with ASS and β-Blockers.

### PCI

In our sample, only four patients were transferred for invasive investigation within the first seven to nine days. Another two patients received PCI in the first month after AMI. Altogether, interventional treatment was performed in six cases within one month after AMI.

### Fibrinolytic treatment

For the study period, 82 patients (61% of the study population) were treated with the main diagnosis STEMI. 57 (69.5%) of them (34 men and 23 women) were admitted to a hospital within the first 12 hours from symptoms onset (see Table 3). In the medical record of one man, contraindications for conducting fibrinolytic treatment were noted. One man and two women were admitted with cardiac shock and died away within a short time after admission. According to the information obtained from the inpatient charts, 53 subjects (32 men and 21 women) were eligible for fibrinolytic treatment. Fibrinolytic reperfusion therapy with Alteplase was performed in 32% (N = 17). Lysis was applied mainly in men (N = 13; 41% of the eligible male population). Only 19% of the eligible women received this treatment. 53% of the patients were < 65 years old.

### In-hospital and early post-discharge mortality

10 out of the sample of 134 patients died during inpatient treatment (7.5%), with male mortality being 3.4% (N = 3), and female mortality being 15.6% (N = 7). Nine of the deceased patients were > 65 years of age. Within 14 days, one more patient died at home.

### Length of stay (LOS)

The median LOS was 8 days (mean 8.5, SD ± 3.3) for the total population. For males, the median LOS was 9 days (SD ± 3.4) and for females 7.6 days (SD ± 2.9). The LOS of the fibrinolysis group did not differ from the total population with a median of 8 days (mean 8.7, SD ± 3.3). The mean LOS according to age was 8.8 days (SD ± 3.1) for patients aged 26 to 64 years and 8.35 days (SD ± 3.4) for patients ≥ 65 years.

## Discussion

Our study shows that most patients with AMI treated in the Stara Zagora region in Bulgaria accessed hospital treatment with a time delay and did not receive the full extent of internationally recommended treatment. Within our study group, patient characteristics did not influence length of stay in the hospital.

### Population

The ratio NSTEMI:STEMI (1:1.58) and female:male (1:1.98) in our study population complies with the results of a survey conducted in 2001–2003 in another Bulgarian region – the Sofia district [12]. The male-female patient ratio is close to that of other European countries [13,14]. The medical histories, considering MI and AP in the past, as the distribution of co-morbidities (hypertension and diabetes) in the study region correspond in terms of frequency to those of other regions of the country [12].

### Pre-hospital time delay

It is obvious that "pain to door" time for AMI in the Stara Zagora Region, as measured by our study, is longer compared to a number of international multicenter trials [13]. A time-trend analysis of this subject is not possible, as no previous data has been published for the region of Stara Zagora.

In contrast to previously reported studies [15,16], in the region of Stara Zagora an age ≤ 64 years or higher education levels (college or high school) were associated with a significantly longer pre-hospital time delay (see Table 4). Explanations for these findings might be the insufficient training of the emergency-care staff concerning ischaemic heart attacks in younger subjects, insufficient medical knowledge of the public and mistrust in the health care system especially in higher educated patients.

The fact that we could not establish a significant relation between pre-hospital time delay and distance between hospital location and patient's residence as well as manner of arrival to hospital backs the assumption that pre-hospital time delay could be determined by factors related to the health care system itself:

First of all, the public in general is not sufficiently informed about the initial symptoms of MI. Although many people do know that chest pain is a presenting symptom of MI, they are uninformed about associated symptoms such as pain in the arm, pain of the lower jaw, shortness of breath, nausea, etc., and are unaware of the fact that it is necessary to seek medical assistance within the first 20–30 minutes. The latter was confirmed by one of the results of our interview study showing that 60% of the patients self-evaluated their hospital admittance as being within an optimal time interval [17].

Secondly, the Bulgarian population's negative opinion of the health care system combined with the fact that admittance to a hospital has become very complicated in recent years resulted in inhabitants of smaller villages in particular preferring to wait out the symptoms at home or go to their physician in his or her consultation hours, which may be after two or three days. As telephone communication in the region of Stara Zagora is good, over 80% of the inhabitants have a telephone in their home. Consequently, calling the emergency services should not be a problem for most of the population. However, it is not always ensured that a call also elicits a response.

### Treatment with Heparin

During the acute care period, heparinization was performed in 98.3% of the cases. Compared with data from the Global Registry of Acute Coronary Events (GRACE) [18], the rate is relatively higher (98% for the Stara Zagora region versus 86.0% for the GRACE population). A possible explanation of this finding could be the limited possibilities for PCI treatment as well as for fibrinolysis.

### In-hospital medical treatment within the first 24 hours and at discharge

Optimal conservative therapy of AMI includes at least the use of β-Blockers and ASS, the use of Angiotensin-Converting Enzyme ACE-Inhibitors as well as cholesterol status assessment and management with lipid-lowering drugs [19].

Administration rates for in-hospital medical treatment during the first 24 hours from the Stara Zagora region for ASS are similar to, and for β-Blockers higher than the figures reported by Jencks et al. [20] from the USA. Rates for both ASS and β-Blockers are lower than those reported from western European countries (93.8% for ASS) [14] as well as those from the GRACE population (98.4% for ASS and 80% for β-Blockers) [18]. During their hospitalization, patients in the age group ≤ 65 years were less likely to receive ASS and β-Blockers than the patients in the same age group from the CRUSADE study (77% versus 93% for ASS and 71% versus 80% for β-Blockers) [8].

Our study shows the use of conservative medication at discharge in patients with AMI being close to the figures reported by Jencks et al. [20] for ASS, higher for β-Blockers and lower for ACE-inhibitors. A comparison with data from western European countries shows that the administration rates for the same drugs in the Stara Zagora region are lower (91% for ASS, 80.5% for β-Blockers and 79.5% for ACE-Inhibitors) [14]. The use of lipid-lowering drugs at discharge from Bulgarian hospitals is also lower than the figures from the Euro Heart Survey of ACS [21]. In contrast to findings from other studies [22,8], the women and older patients from the Stara Zagora region were more likely to receive ASS, β-Blockers and ACE-Inhibitors or AR-Blockers. The use of lipid-lowering drugs was somewhat higher in women and patients ≤ 65 years of age.

### Fibrinolytic treatment

In the Stara Zagora region, fibrinolysis under hospital conditions can be performed in five of the six hospitals. One of the reasons for the limited application of fibrinolytic reperfusion, hospital admission delay, has already been discussed.

Another reason may be associated with hospital funding in Bulgaria. In 2004, the National Health Insurance Fund (NHIF) reimbursed € 1,175 for "ACS with persistent ST-elevation and fibrinolysis", which includes only Alteplase or Reteplase applications [11]. The estimated price at which hospitals may have bought the most commonly marketed drug Alteplase was € 870 for the course of one treatment. From the reimbursement amount (€1,175), 40% (€ 470) are used for personnel remuneration. Thus, a mere € 705 remains for laboratory and instrumental diagnostics, lytic treatment, other medication, board and lodging. Reteplase, which is more expensive at € 1,023, is rarely used in Bulgaria. As a consequence of deficient financing, physicians face limitations in decision-making and fibrinolysis in ACS with persistent ST-elevation is applied relatively rarely and depends more on the hospital's actual budget situation than on patients' medical needs.

Reperfusion with Streptokinase (SK), which was applied in Bulgaria until 2001 is not reimbursed by the NHIF. Despite the fact that SK-administration could be associated with a rapid rise in neutralizing antibodies, making repeated administration impractical [3,23,24], the treatment with SK is associated with a lower risk for non-cerebral and cerebral bleeding complications compared to Alteplase [25]. Besides, SK is significantly cheaper [3]. In Bulgaria, the price of 1.5 million units of SK (the common fibrinolysis dose) is about € 164.0.

According to up-to-date evidence, the implementation of pre-hospital intravenous fibrinolytic reperfusion therapy should be considered when the time needed to reach an inpatient health care facility is estimated to exceed 60 minutes [26]. Unfortunately, in the region of Stara Zagora, the initiation of treatment under outpatient conditions does not take place for a number of reasons – lack of thrombolytic drugs, lack of training and poor conditions during transportation.

### PCI

In Bulgaria and in the Stara Zagora region in particular, PCI facilities are very limited. Although ischaemic heart disease represents one of the most common reasons for death in Bulgaria, the health care system does not provide the facilities to perform the adequate number of invasive interventions. The medical facilities in Bulgaria are able to provide 254 invasive investigations and 124 interventional treatment procedures per one million inhabitants per year. Considerable investments in equipment, technology and training are necessary in order to meet the population's needs. For this reason, the fibrinolytic reperfusion therapy represents the most common reperfusion strategy for the medical management of STEMI in Bulgaria and in the Stara Zagora region.

### Mortality

The mortality rate at Stara Zagora hospitals is similar to the figures of the GRACE trial [18], as well as data of other surveys [7]. The gender distribution shows female mortality being somewhat higher than male mortality. The common ratio is about 2:1 [27], which corresponds to the data of another Bulgarian region [12].

In our study, the average age of the deceased women was 72.6 years versus 76.3 for men. Four of the females were admitted within the first two hours after symptoms onset versus one man admitted in the same time interval. Only two women did not need resuscitation and ventilation in the first hours after admission. All of the females had hypertension in the medical history.

These results, as well as the lower use of fibrinolytic therapy among eligible women than men, raise the possibility of gender differences in AMI treatment in Bulgaria. The lack of knowledge or sensitivity towards gender specific symptoms and risk factors related to IHD, especially within the outpatient setting, may be explained by a lack of training of medical personnel in this regard. Our findings regarding gender specific mortality in ACS patients need further investigation.

### LOS

Regardless of the treatment actually provided, the health care service purchaser NHIF limits the duration of in-hospital treatment in Bulgaria to eight days. The comparison of the LOS in Stara Zagora hospitals with data from other countries, especially from Western and Central Europe shows that patients in Stara Zagora with ACS have shorter median stays. The mean hospital stay with lysis is 13 ± 5 days in the PRAGUE-2 trial [28] as opposed to 9 ± 3 days for the Stara Zagora region.

### Generalisation of the study results

The survey we conducted represents the management of AMI for 5% of the Bulgarian population. The results obtained may be representative for most regions, with the exception of the national capital. Currently, the health care system of Bulgaria cannot provide sufficient PCIs, which is the reason why fibrinolytic reperfusion and conservative drug therapy remain the methods of choice for ACS treatment. Internationally, fibrinolytic therapy demonstrated remarkable life-saving effects never observed before and at the same time represented the beginning of "millionaire medicine" [26].

Regardless of the fact that the resources allocated by the NHIF do not cover the expenses, the fund has selected Alteplase or Reteplase to be the only fibrinolytic drugs in Bulgaria. Other Central European countries, which, like Bulgaria, are reforming their health sector, continue to use Streptokinase routinely, although they obviously have better funding capacity [28]. In addition, Streptokinase is still used as the treatment of first choice in low-risk patients in the UK, Australia, New Zealand and the Netherlands [26]. Therefore, revising the NHIF disease management program for STEMI and tailoring the reperfusion strategy in a way which acknowledges the financial situation of the Bulgarian health care system should be the first step towards improving AMI treatment performance in Bulgaria. If the NHIF considered the possibility of Streptokinase treatment, a larger number of patients could receive fibrinolytic reperfusion therapy leading to improved outcomes of inpatient management of STEMI.

## Conclusion

In spite of the efforts of health care policy-makers to improve the system, the results of the health sector reforms in Bulgaria, particularly within the field of cardiology, still show much room for improvement. Quality of care of patients with AMI in Bulgaria is still far from the evidence-based recommendations. 18 years after the breakdown of the former socialist Semashko system, the Bulgarian health care system is still not able to provide state of the art health care to decrease AMI mortality. To achieve the goal of reducing cardiovascular disease mortality by introducing up-to date management of ACS, Bulgaria will have to provide financial and human resources which are currently neither available in the region of Stara Zagora nor in the rest of the country.

## Study limitations

Our study is limited in a number of ways. First of all, the data were collected retrospectively from inpatient records. In Bulgarian hospitals, methodological standards for the contents of inpatient records concerning medical history, smoking habits, body-mass index, and indications for treatment have not been introduced. Thus, we were not able to encompass all important patient characteristics in our study.

Of particular concern is the issue of the discharge diagnosis. Despite the fact that the NHIF has implemented diagnostic criteria for AMI (STEMI and NSTEMI) and the medical staff do their best to fulfil them in order to receive the reimbursement fee for patients' treatment, there is a tendency of "up-coding" in some cases.

The exact pain-to-door time was only available for 74% of the study population. The relationship between pre-hospital time delay and socio-demographic factors is included in the study, but the item needs further investigation in a larger population.

Similarly, the age and sex differences in treatment at discharge and in mortality need to be investigated in larger populations.

Notwithstanding the limitations mentioned above, our work depicts the inpatient care for patients with ACS in a representative region of Bulgaria. In addition, the study brings forward some of the obstacles for improving quality of health care for AMI which is the most important burden of the health care system and the entire Bulgarian society.

## Competing interests

The authors declare that they have no competing interests.

## Authors' contributions

MGI conceived and designed the study and drafted the manuscript. KK performed data analysis and revised the manuscript critically for important intellectual content. MG contributed to conception and design of the study and revised the manuscript critically for important intellectual content. All authors read and approved the final manuscript.

## Pre-publication history

The pre-publication history for this paper can be accessed here:


